# Therapeutic Agents for the Treatment of Temporomandibular Joint Disorders: Progress and Perspective

**DOI:** 10.3389/fphar.2020.596099

**Published:** 2021-01-29

**Authors:** Mengjie Wu, Jingyi Cai, Yeke Yu, Sihui Hu, Yingnan Wang, Mengrui Wu

**Affiliations:** ^1^ The Affiliated Hospital of Stomatology, School of Stomatology, Zhejiang University School of Medicine, and Key Laboratory of Oral Biomedical Research of Zhejiang Province, Hangzhou, China; ^2^ State Key Laboratory of Oral Diseases and National Clinical Research Center for Oral Diseases and Department of Orthodontics, West China Hospital of Stomato-logy, Sichuan University, Chengdu, China; ^3^ Shanghai Key Laboratory of Stomatology and Shanghai Research Institute of Stomatology, National Clinical Research Center of Stomatology, Department of Oral Surgery, Shanghai Ninth People’s Hospital, Shanghai Jiao Tong University School of Medicine, Shanghai, China; ^4^ College of Life Sciences, Zhejiang University, Zhejiang, China

**Keywords:** temporomandibular joint, pain management, intra-TMJ injection, mandibular dysfunction, temporomandibular joint disorder

## Abstract

Temporomandibular joint disorders (TMD) are a common health condition caused by the structural or functional disorders of masticatory muscles and the temporomandibular joint (TMJ). Abnormal mandibular movement in TMD patients may cause pain, chronic inflammation, and other discomfort, which could be relieved by a variety of drugs through various delivery systems. In this study, we summarized commonly used therapeutic agents in the management of TMD as well as novel bioactive molecules in preclinical stage and clinical trials. The emerging therapy strategies such as novel intra-TMJ delivery systems and implants based on tissue engineering are also discussed. This comprehensive review will strengthen our understanding of pharmacological approaches for TMD therapy.

## Introduction

Temporomandibular joint disorders (TMD) are complex clinical problems related to the dysfunction of the jaw joint and masticatory muscle. TMD occurs more often in women than in men aged 20–40 years old. Its estimated that the prevalence of TMD in children and adolescents varies from 6% to 68%, considering the inconsistency among diagnostic criteria and clinical examination adopted (Chang and Israel, 2005). Epidemiological studies revealed that muscle disorders, disc displacements, and other joint disorders are the most common symptoms of TMD (Osiewicz et al., 2018).

The occurrence and progress of TMD are associated with the damage or structural alterations of the temporomandibular joint (TMJ). Pain, malocclusion, constrained and abnormal jaw motion, and joint noises are the most common chief complaints of patients with TMD. Concomitant headaches and sleep disturbances exacerbate the sharp decline of patients’ life quality. Fortunately, pharmaceutic therapies have been vastly used in the past 25 years to relieve the syndrome of TMD. The most effective pharmacological agents used to treat TMD include nonsteroidal anti-inflammatory drugs (NSAIDs), opioids, corticosteroids, anxiolytics, muscle relaxants, antidepressants, and anticonvulsants (Ouanounou et al., 2017). We performed an electronic search of PubMed database between 2011 and July 2020 with focus on TMD, defects, and various types of novel drugs/delivery systems. Inclusion criteria were broad, and exclusion criteria were studies that were not published in English, published before 2011, or not related to TMJ diseases or injury. Based on the included published papers, we reviewed the approved pharmacologic agents for patients with TMD, as well as novel therapy strategies in preclinical researches and clinical trials. The state-of-the-art review presents an overview of the therapy choices for treating TMD, which may strengthen our understanding of the risks and benefits of these therapeutic agents.

## Clinical Classifications and Management of TMD

### Research Diagnostic Criteria of TMD

Any diagnosis of TMD comes from the evaluation of signs and symptoms. TMD has a variety of classification and nomenclature systems, including the Research Diagnostic Criteria for TMD and the American Academy of Oral and Maxillofacial Pain classification. Many modified taxonomies have been reported recently, such as a new surgical classification for TMD and a proposed classification of TMD in edentulous patients (Dimitroulis, 2013; Alzarea, 2015). Peck et al. expanded and summarized the taxonomy of TMD derived from reliable diagnostic and consensus-based diagnostic criteria (Peck et al., 2014) as shown in Table 1, which presents a state-of-the-art classification of TMD (Schiffman et al., 2014).

**TABLE 1:** Expanded taxonomy of TMD (Peck et al., 2014).

I. TMD	1.A: Joint pain (arthralgia and arthritis)
	1.B: Joint disorders (disc disorders, hypomobility disorders other than disc disorders, and hypermobility disorders) Disc disorders: disc displacement with reduction and disc displacement with reduction with intermittent locking Disc displacement without reduction with limited opening Disc displacement without reduction without limited opening hypomobility disorders other than disc disorders
	1.C: Joint diseases (degenerative joint disease, systemic arthritides, condylysis/idiopathic condylar resorption, etc.)
	1.D: Fractures
	1.E: Congenital/developmental disorders (aplasia, hypoplasia, and hyperplasia)
II. Masticatory muscle disorders	2.A: Muscle pain (myalgia, tendonitis, myositis, and spasm)
	2.B: Contracture
	2.C: Hypertrophy
	2.D: Neoplasm
	2.E: Movement disorders (orofacial dyskinesia and oromandibular dystonia)
	2.F: Masticatory muscle pain attributed to systemic/central pain disorders (fibromyalgia/widespread pain)
III. Headache	Headache attributed to TMD
IV. Associated structures	Coronoid hyperplasia

### Pharmacological Agents in the Clinical Management of TMD

The etiology of TMD is complex and multifactorial, which is caused by related functional, structural, and psychological factors. TMD include muscle-related myogenic TMD and joint-related arthrogenic TMD. Touche et al. reviewed the evidence on treatment options for pain related to TMD in their Table 1. The myogenic and arthrogenic TMD is different in etiology, pathogenesis, clinical signs, and symptoms; thus, different therapeutic strategies should be employed for their pain management (Gil-Martínez et al., 2018).

**FIGURE 1:**
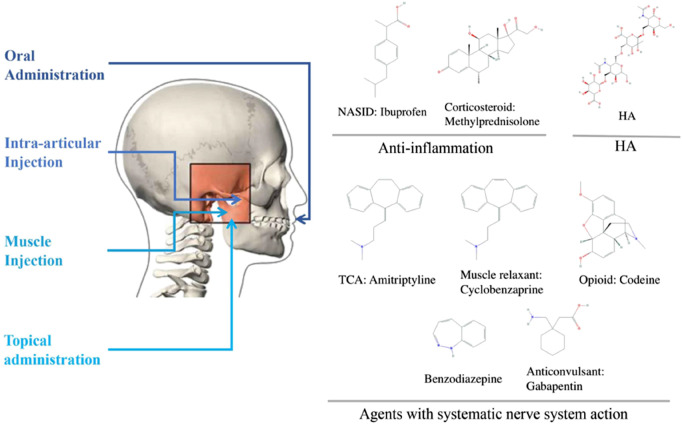
Commonly used agents for treating TMD and their structures.

Pharmacological agents are applied in TMD as a monotherapy or in combination with other approaches, such as physiotherapy, occlusal splint, and surgical intervention. Patients with chronic TMD have frequently overlapping pain conditions of systematic disease. Compared with localized TMD, they are more likely to suffer from depression and anxiety, sleep poorly, distressed and arguably. Thus, researchers and clinicians have adopted several different drug delivery routes, including oral administration, intra-articular (IA) injection, muscular injection, and topical administration, such as ointment and cream. The following sections describe the indications of each intervention and its potential therapeutic effects for TMD patients. Here, we classified commonly used TMD drugs into four groups, namely, anti-inflammatory drugs, agents with systemic nervous system actions, hyaluronic acid (HA), and glucosamine (GS), and discussed their administration methods accordingly (Figure 1).

#### Anti-inflammation Drugs

In TMD, inflammation is observed along with pain and cartilage degeneration. Therefore, drugs, such as NSAIDs and corticosteroids, which inhibit the release of inflammatory cytokines, are prescribed in TMD. The compelling analgesic and anti-inflammatory effects of NSAIDs, such as their ability to block prostaglandin synthesis by inhibiting cyclooxygenase, made this type of drugs among the first-line options for mild to moderate inflammatory pain in patients with TMD (Hersh et al., 2008). However, most NSAIDs for TMJ pain, including ibuprofen and naproxen, are given through oral administration, which might have side effects, such as the exacerbation of hypertension, gastrointestinal effects, and the worsening of renal function. The IA application of tenoxicam might be more effective than orally administered drugs in terms of anti-inflammatory and analgesic effects. Corticosteroid is another potent anti-inflammatory drug that can inhibit the release of arachidonic acid, from which prostaglandins and leukotrienes are derived. Glucocorticoids, usually diluted with local anesthetic, are most widely utilized for IA injection to patients with TMD for its potential advantages, such as its safe use, less systemic exposure, and few side effects (de Souza et al., 2012).

#### Agents with Systemic Nervous System Actions

Antidepressants, muscle relaxants, opioids, and anti-convulsants, which suppress the responses of the peripheral or central nervous system (CNS), are commonly employed to relieve TMJ pain or muscular spasm. Among various antidepressants, tricyclic antidepressants and serotonin norepinephrine reuptake inhibitors seem to be the most effective for chronic pain, whereas selective serotonin reuptake inhibitors may reduce orofacial pain (Finnerup et al., 2010). Muscle relaxants are prescribed to relieve myofascial pain in TMD. Cyclobenzaprine is one of the most commonly prescribed muscle relaxants in TMD that may inhibit descending serotonergic pathways in the spinal cord by acting on 5-HT2 receptors. Most muscle relaxants function systematically in the CNS and alleviate pain by releasing the suppressive tonic flow of nerve impulses, which are later transmitted to voluntary muscles (Bal Kucuk et al., 2014). Opioid peptides, as a classical analgesia, mimic endogenous opioids and target endogenous μ-opioid receptors to inhibit the transmission and sensation of pain. Considering the uncontrollable side effects and potential abuse of opioids, their prescription is not suggested unless all other treatment options are intractable (Yip and Oettinger, 2020). Intramuscular morphine elevates mechanical pain threshold and tolerance in the masseter in TMD with myofascial pain with little systemic effect in clinical trials (Yip and Oettinger, 2020).

#### Hyaluronic Acid (HA)

Along with glucocorticoids, HA is commonly administered through IA injection for TMD treatment. HA is derived from the natural component of the synovial fluid and articular cartilage; thus, it can restore the viscoelasticity of the synovial fluid in an inflamed joint and has lubrication, shock absorption, and joint protection effects (Evans et al., 2014). Moreover, HA can interact with different receptors to exert effects in anti-inflammation, cartilage synthesis, and chondroprotection (Webb and Naidoo, 2018). The pain alleviating effects of HA and glucocorticoid seem nearly the same, although the repeated IA injection of glucocorticoids may lead to degenerative changes in the joint, which is not seen during the use of HA (Heir, 2018). Moreover, intramuscular injection of collagen has also been shown to be an efficient method for reducing myofascial pain within masseter muscles (Nitecka-Buchta et al., 2019a).

#### Glucosamine (GS)

GS is the metabolic precursor for extracellular matrix synthesis and has been reported to inhibit cartilage decomposition and promote proteoglycan synthesis (Wu et al., 2013). As an over-the-counter medicine, GS appears to be safe, and oral GS is usually prescribed as an adjunct to HA injection in treating TMJ osteoarthritis. These traditional therapies are mostly empirical treatment without potent evidence to support their effectiveness and coherence.

## Novel Molecular Agents for TMD Treatment

**FIGURE 2:**
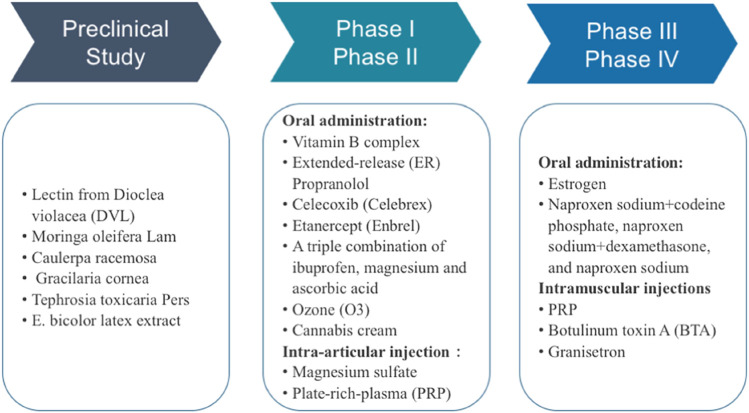
Stages of preclinically and clinically studied agents for TMD therapy.

Traditional pharmacological agents for TMD are effective but have unavoidable side effects and unwanted interaction with commonly used drugs. Thus, novel and safer therapeutic strategies are still in great demand. In the past decades, great progress has been achieved to discover new chemical entities for the prevention and treatment of various TMD syndromes. Among these prophylaxis, plant-derived compounds and crude extracts stand out, and other medical products, such as ozone and platelet-rich plasma (PRP), were also tested in clinical study (Daif, 2012; Rahimi-Movaghar and Eslami, 2012; Celakil et al., 2019). Drug repurposing strategy was as well employed in clinical trials to evaluate the efficacy of FDA-approved anti-arthritis drugs, such as celecoxib, for TMD therapy or the new combinations of existing TMD drugs. In this part, we summarized the treatments used in preclinical research and clinical trials as shown in Figure 2.

### Bioactive Compounds in Preclinical Studies

Naturally occurring products have long been reliable and abundant resources for drug development (Li and Vederas, 2009). Nature derivatives with anti-inflammatory and analgesic effects hold great promise for application in TMD medication. Lectins, extracts from plants and algae, sulfated polysaccharides (SPs), and isolated compounds, such as terpene, are among the widely explored nature-derived agents, and a latest review with relatively strict inclusion criteria provided a hint to this field but without elucidating their mechanisms. Our understanding of the side effects and proper dosage of these natural products are lacking; thus, preclinical research on these drugs should be highlighted. TMD rat models provide a good platform to test these drugs. In a rat disease model, TMD could be stimulated through the IA injection of various agents, such as formalin, capsaicin, serotonin, carrageenan, and mustard oil. Formalin, as an agonist of transient receptor potential ankyrin subtype 1 protein (TRPA1), which is a nonselective cation channel expressed in pain-sensing neurons and a promiscuous chemical sensor involved in sensory hyperreactivity in visceral organs, has been used to establish a stable model for an orofacial nociception study (Coura et al., 2017; Melo et al., 2017). Capsaicin, as a derivative from red pepper, binds to transient receptor potential cation channel subfamily V member 1 (TRPV1) to activate sensory terminals and stimulate burning pain after injection (Ribeiro et al., 2020). The nociception induced by serotonin is realized in a rather indirect way on primary neurons via the activation of sympathomimetic amines and the synthesis of prostaglandin (Ribeiro et al., 2020; Oliveira-Fusaro et al., 2012). Zymosan, a polysaccharide derived from yeast cell walls, could induce severe and erosive arthritis and severe pain once injected (do Val et al., 2014). Carrageenan and mustard oil could induce plasma protein extravasation and leukocyte migration; mustard oil simultaneously causes in neuron depolarization and neurogenic inflammation, whereas carrageenan represents the non-neurogenic one (Clemente-Napimoga et al., 2019). The vibrissal pad injection of complete Freund’s adjuvant (CFA) has also been developed as a reliable model for TMD study (Luo et al., 2020). Researchers observe the duration of nociceptive behaviors, such as scratching orofacial regions, reflex response, or mechanical allodynia, to assess the severity of pain and measure TMD relief. Samples, including synovial fluid and periarticular tissues, are also obtained to test for inflammatory factors, granulocyte infiltration, and vascular permeability. However, although these novel therapeutic agents might open avenues to treat TMD, their efficacy and probable toxicity profiles are still waiting to be exploited in the future for better and safer application.

#### Lectins

Lectins are a heterogeneous group of proteins that possess noncatalytic domain binding sites and exhibit specific and reversible binding ability to simple sugars or complex carbohydrates. Considering the wide distribution and reliable medical effects of lectins as anti-inflammation factors, several plants have been tested for lectin extraction and TMD treatment. First, lectin from *Dioclea violacea* (DVL) was verified to have high affinity with ligands, such as α-methyl-D-mannoside, N-acetyl-D-glucosamine, and core 1 sialyl Lewis X (core 1-sLeX). In TMD rat models induced by either carrageenan or mustard oil as non-neurogenic and neurogenic types, respectively, the application of DVL impaired the inflammation process and may serve as a promising strategy for TMD treatment (Clemente-Napimoga et al., 2019). Mechanically, leukocytes bind to endothelial cells to enable their extravasation and then transfer to the inflammation spot, in which the appropriate interaction between selectins and their ligands is needed (Nourshargh and Alon, 2014). DVL, as a selectin, could bind and block the core 1-sLeX on the surface of leukocytes, inhibit the ligand–selectin bound to endothelial cells, decrease intercellular adhesion molecule-1 expression, and thus inhibit leukocytes from rolling and binding to the surface of endothelial cells and the subsequent inflammatory process. Lectin from *Abelmoschus esculentus* exert antinociceptive and anti-inflammatory effects on zymosan-induced TMJ pain partially by inhibiting inflammatory cytokines, such as TNF-α and IL-1β, and by depending on the integrity of heme oxygenase 1 (HO-1), whose reduction would facilitate the inflammation process (Freitas et al., 2016). Meanwhile, the activation of central δ and κ opioid receptors serve as another explanation of its antinociceptive effect (Alves et al., 2018). Notably, lectin from *Caulerpa cupressoides* has a role in TMJ related to TNF-α and IL-1β inhibition but is independent of the HO-1 pathway or opioid receptors (da Conceição Rivanor et al., 2014). Lectin isolated from the seeds of *Artocarpus incisa* L., such as frutalin, acts as an inhibitor of orofacial nociception in acute and chronic pain, which is mediated by TRPA1, TRPV1, and transient receptor potential melastatin 8 receptors (Damasceno et al., 2016). As information mediators, lectins play an essential role in inflammation and pain; thus, their importance is worthy of further exploration.

#### *Moringa oleifera* Lam

*M. oleifera* L*.* is a folk recipe in the tropics utilized for diversified medical purposes, including anti-inflammation. Although *M. oleifera* has considerable therapeutic effects, its wide application is retarded because their properties lack elucidation. Researchers have developed a set of derivations of extractions from *M. oleifera* and tested the toxicological index of each molecule, which confirmed that MC-D7, MC-D9, and MC-Hl are safe analogs. Furthermore, hypernociception is reduced comparatively by the application of these three molecules in a rat model of TMD, which may be explained by their mediation on the inflammatory factors, along with the effect on the NF-κB signaling pathway by suppressing the degradation of IκB. Reduction of the vascular permeability was also observed in this study (Dos Santos et al., 2018). Another *in vitro* program proved that moringin is a potent agonist of the TRPA1 channel (Borgonovo et al., 2020). Thus, the application of these analogs would benefit TMD treatment in a relatively safe way with few side effects.

#### *Tephrosia toxicaria* Pers

*T. toxicaria* P. is also perceived as a folk recipe in Amazonian countries for pain and inflammation alleviation. *T. toxicaria* P. relieves inflammatory hypernociception in the TMJs of rats hypothetically depending upon the integrity of the HO-1 pathway (do Val et al., 2014). Another study in mice provided evidence that its anti-inflammatory activity is related to the inhibition of pro-inflammatory cytokines, such as TNF-α and IL-1β, as well as the NO-dependent inhibition of leukocyte recruitment (Martinez et al., 2013).

#### *Euphorbia bicolor* Latex Extract

Patients with TMD have remarkably elevated oxidative stress biomarkers and reduced total antioxidant capacity, which suggest the potential effects of novel therapeutics with antioxidant and free radical scavenging activities (de Almeida and Amenábar, 2016). Recently, corresponding analgesia was observed with the injection of *E. bicolor* latex extract in a rat model of orofacial pain. In this process, its role in antioxidant activities matters. Basically, the extract downregulated the level of advanced oxidation protein product (AOPP), leading to fewer reactive oxygen species (ROS) produced via Nox4, which would be followed by a consequential decrease in activation of TRPV1 as the peripheral pain generator (Basu et al., 2019). The molecule provided a cure for both acute and chronic pain and functioned independently of the opioid receptors, which indicate its medical potential (Basu et al., 2019).

#### SPs from Marine Algae–derived Products

Apart from terrestrial resources, marine algae–derived products also represent a set of promising agents, among which the abundant repertory of SPs stands out in the pharmaceutical industries for TMD treatment. Here, we listed three SPs available for TMD management, namely, the purified SPs from *Caulerpa racemosa* (Cr), the polysaccharidic fraction I of *Gracilaria cornea* (Gc-FI), and SPs (Fraction F II) from red seaweed *Solieria filiformis*. SPs from Cr and Gc-FI share common effects on inflammation-related cytokines, that is, Cr reduces TNF-α and IL-1β, whereas Gc would do the same downregulation and increase the release of IL-10 (Coura et al., 2015). However, discrepancies were also observed. First, pre-treatment with Cr alleviates the TMD induced by serotonin, but the same application of Gc-FI failed to provide an improvement. This finding suggests that the inhibition of sympathomimetic amines and prostaglandin synthesis, which are found in Cr but not in Gc-FI, are required for the treatment to work. Second, hypernociception in animal models requires different neurotransmitters and neuromodulators, such as nitric oxide (NO) and carbon monoxide (CO), which represent the NO/cyclic guanosine monophosphate/protein kinase G/ATP-sensitive potassium channel (NO/cGMP/PKG/K + _ATP_) pathway and the heme oxygenases (HO)/CO/cGMP/PKG pathway, respectively, to regulate nociception. Gc-FI is dependent on NO and CO, whereas Cr only relies on the HO-1 pathway and its functionality not influenced by the integrity of NO (Ribeiro et al., 2020; Coura et al., 2017). Last, Gc-FI also depends on the activation of opioid receptors to exert its antinociceptive effect, which was also confirmed in SPs from *S. filiformis* (Araújo et al., 2017). Rather than being selective, SPs from *S. filiformis* interact with all the three opioid receptors in the TMJ hypernociception of rats, such as morphine, and thus may serve as a relatively safer alternative for opioids.

#### Terpene

Terpene is a large category of structurally diverse molecules and includes primary and secondary metabolites. As the main constituent of essential oils, the demand for terpene in the cosmetic and pharmaceutical industries keeps on increasing for years (Melo et al., 2017; Paramasivan and Mutturi, 2017). (−)-α-bisabolol (BISA), a sesquiterpene widely used commercially as a cosmetic ingredient, gained the classification as “generally regarded as safe” from the FDA. Several studies have confirmed its efficacy in TMD pain as an antagonist to TRPA1 and are associated to TNF-α but not IL-1β reduction (Barreto et al., 2016). BISA also appears to be effective against trigeminal neuropathic pain for its role in central sensitization after the transection of the infraorbital nerve (Melo et al., 2019). Notably, the efficacy of the oral or topical administration of BISA suggests its clinical importance as a promising adjuvant for orofacial pain treatment. Interestingly, a clinical research already verified that BISA mouthwash contributes to pain relief and healing in postoperative maxillofacial surgeries (Amora-Silva et al., 2019). Similarly, eucalyptol, a natural monoterpenoid used as the main active component of several essential oils, including that extracted from Tasmanian bluegum, has antinociceptive effects via its antagonism to TRPA1 (Melo Júnior et al., 2017). More research into the therapeutical roles of terpenes would be of great importance, given that the commercialization of terpene is already relatively mature (Paramasivan and Mutturi, 2017).

#### Dietary Treatment: Cocoa, Grape Seed Extract (GSE), and Purple Corn Extract

Apart from chemical medication, alteration in food and nutrition would be an economic and safe adjuvant to TMD therapy. Studies on cocoa, GSE, and purple corn extract provided satisfying results; thus, these foods are likely to be applied clinically. Cocoa, as a widely accepted food, contains various phytochemicals, including polyphenols, which suggest its possible effects in anti-inflammation, antioxidation, and pain relief (De Feo et al., 2020). Rats fed by a cocoa-enriched diet for 2 weeks showed better resistance against trigeminal nerve activation induced by acute (capsaicin injection to eyebrow regions) and chronic (CFA injection to TMJ) stimuli. Mechanically, cocoa diet would contribute to an increased basal levels of mitogen-activated protein kinase phosphatase (MKP)-1 and MKP-3 in neurons, which would then negatively regulate mitogen-activated protein kinases, phosphorylated p38 (P-p38), and P-ERK. Meanwhile, the blockage of calcium channel activity would inhibit calcitonin gene-related peptide (CGRP) and enhance the anti-inflammatory effects of cocoa. Nitric oxide synthase (iNOS) in trigeminal ganglia neurons and the production of NO decreased in the group of cocoa-fed rats (Cady and Durham, 2010). Another dietary experiment with GSE also provided us with a hint for TMD therapy. Rats fed with a GSE-enriched diet for 14 days had an elevated basal expression level of MKP-1 in neurons, as well as glia in the trigeminal ganglia and nucleus caudalis. The increased basal expression of glutamate aspartate transporter in the spinal glia and the suppression of neuropeptide CGRP in spinal neurons also decrease neuronal excitability. The suppressed expression of P-p38, OX-42, and glial fibrillary acidic protein is achieved by dietary intervention in response to chronic inflammation induced by CFA (Cady et al., 2010); thus, the resistance to induced pain is stronger. Similarly, rats that drank purple corn extract for 14 days showed better resistance to orofacial allodynia. Purple corn, compared with its isogenic yellow corn, is enriched with anthocyanins. Apart from the traditional target of TMD, such as neurons, purple corn extract reduces trigeminal macrophage infiltration, which represents the hallmark of inflammatory trigeminal pain (Villa et al., 2010). Meanwhile, the shift in microglia cell polarization to a neuroprotective phenotype, such as a thinner and longer shape, accompanied by elevated anti-inflammatory property, was verified as well (Magni et al., 2018).

#### Resveratrol (RSV)

With deep understanding of the human body as a systematic organism, the relationship between gut microbiome perturbation and TMD also gained attention, and researchers hypothesized that maintaining gut microbiome may be a novel approach for treating pain. RSV can be extracted from various plant products, especially grape skins and red wine, and possibly bridge the gut microbiome and TMD. In CFA-induced inflammatory TMD models, short-chain fatty acids (SCFAs) in the gut and relevant bacteria, such as Bacteroidetes and Lachnospiraceae, decreased, and by reversing this CFA-caused reduction and restoring the normal gut microbiota, RSV can resist the inflammatory process and maintain the integrity of the blood–brain barrier (BBB), block the activation of microglia, and inhibit the release of TNF-α in the spinal trigeminal nucleus caudalis (Sp5C) (Ma et al., 2020). This interaction between gut bacteriome and TMD deserves attention, and the trial of RSV should be an interesting starter.

Natural bioactives have provided relief to millions before the development of modern medicine and represent a precious and enormous repertory for drug exploration. Studies aiming to elucidate the characteristics of these products bear significance for pharmacology and show their effect on curing TMD. Unfortunately, in most studies mentioned above, the therapeutic effects were tested in acute models of inflammation in TMJ, whereas the TMD was presented with a chronic progress. Table 2 summarizes the progress of natural-derived bioactives and their potential pharmacological effects. For further application, chronic models should be established and utilized to explore their potential relief to TMD. Meanwhile, the dosage and toxicology should be further elucidated. Strategically, given their sharing of target with the HO-1 pathway, cytokine factors, and TRPA1 and TRPV1 receptors, the efficacy among novel molecules and with traditional remedies should also be displayed. From another point of view, novel drugs as dietary therapy such as cocoa, topical application of BISA, and RSV targeting gut microbiome suggest that combinational remedy should be considered with these molecules as potent supplements.

**TABLE 2:** Summary of novel treatments for TMD.

Ref and trails	Novel molecule	Intervention	Efficacy	Mechanism
Clemente-Napimoga et al. (2019)	*Dioclea violacea* lectin	Intravenously injection	Carrageenan/mustard oil induced: anti-inflammatory	1) Suppressing ICAM-1 2) Blocking the ligands on leukocyte
Alves et al. (2018)	*Abelmoschus esculentus* lectin	Intravenously injection	Zymosan-induced: antinociceptive and anti-inflammatory; Formalin-induced: central antinociceptive	1) Depending on HO-1 pathway 2) Inhibiting TNF-α and IL-1β 3) Activation of δ and κ opioid receptors but not of μ opioid receptors
da Conceição Rivanor et al. (2014)	*Caulerpa cupressoides* lectin	Intravenous injection	Zymosan-induced: antinociceptive and anti-inflammatory	Inhibiting TNF-α and IL-1β
Damasceno et al. (2016)	Lectin of seeds of *Artocarpus incisa L.*/Frutalin	Intraperitoneally injection	Formalin/glutamate/capsaicin induced: antinociceptive	Modulating TRPA1, TRPV1, and TRPM8 receptors
Dos Santos et al. (2018),Borgonovo et al. (2020)	*Moringa oleifera Lam*	*Per os*	Formalin-induced: antinociceptive and anti-inflammatory (MC-D7, MC-D9, and MC-H) Serotonin-induced: antinociceptive (only MC-H)	1) Mediation on the inflammatory factors 2) Agonist of TRPA1
Coura et al. (2017)	*Gracilaria cornea*	Subcutaneous injection	Formalin-induced: antinociceptive Serotonin (5-HT)-induced: No effect	1) Activating opioid receptor 2) Depending on NO/cgmp/PKG/K + _ATP_ pathway and the HO/CO/cgmp/PKG pathway 3) Inhibiting TNF-α and IL-1β and increasing IL-10
Ribeiro et al. (2020)	*Caulerpa racemosa*	Intravenous injection	Formalin/capsaicin/serotonin-induced: antinociceptive and anti-inflammatory	1) Depending on HO-1 pathway 2) Inhibiting TNF-α and IL-1β
Araújo et al. (2017)	*Solieria filiformis*	Subcutaneous injection	Formalin/serotonin-induced: antinociceptive and anti-inflammatory	1) Activating 3 opioid receptors in the subnucleus caudalis 2) Inhibiting the release of inflammatory mediators in the periarticular tissue
do Val et al. (2014); Martinez et al. (2013)	*Tephrosia toxicaria Pers*	Injection	Zymosan-induced: antinociceptive and anti-inflammatory	1) Depending on HO-1 pathway 2) Inhibiting TNF-α and IL-1β 3) NO-dependent inhibition of leukocyte recruitment
Basu et al. (2019); Barreto et al. (2016); Amora‐Silva et al. (2019); Melo et al. (2019)	*Euphorbia bicolor (Euphorbiaceae) latex*	Injection into the inflamed vibrissal pad.	CFA-induced: antinociceptive and anti-inflammation	1) Down-regulating AOPP, ROS, Nox4, 2) Inactivating TRPV1
	(−)-α-bisabolol (BISA)	Oral administration	Formalin-induced: antinociceptive and anti-inflammation	1) Antagonist of TRPA1 2) Inhibiting TNF-α but not IL - 1β
Melo Júnior et al. (2017)	Eucalyptol	Oral administration	Formalin/mustard oil-induced: antinociceptive	Antagonist of TRPA1
Cady and Durham (2010)	Cocoa	Cocoa-enhanced diet for two weeks	Capsaicin/CFA-induced: antinociceptive and anti-inflammation	1) Elevating the basal level of MKP-1 and MKP-3 in neurons 2) Inhibiting CGRP 3) Decreasing iNOS
Cady et al. (2010)	Grape seed extract	Grape seed extract-enhanced diet for 2 weeks	CFA-induced: antinociceptive	1) Elevating the basal level of MKP-1 in trigeminal nerve 2) Elevating the basal level of GLAST and decreasing that of CGRP in spinal 3) Suppressing expressions of P-p38, OX-42, and GFAP under CFA-induced pain
Magni et al. (2018)	Purple corn extract	Drink purple corn extract for 2 weeks	CFA-induced: antinociceptive	1) Reducing trigeminal macrophage infiltration 2) Shift of microglia cell polarization to a neuroprotective phenotype
Ma et al. (2020)	Resveratrol	Intraperitoneally injection for 4 days	CFA-induced: anti-inflammation	1) Reversing CFA-caused reduction of SCFAs and recovering CFA-decreased Bacteroidetes and Lachnospiraceae in the gut 2) Rescuing CFA-caused BBB leakage 3) Blocking CFA-enhanced microglial activation and expression of TNF-α in the Sp5C
Dehghan (2015) NCT02794922	Vitamin B complex	Oral administration	Stronger analgesic property than vitamin E and diclofenac	1) Protecting cell membrane against peroxidation 2) Enhancing norepinephrine and 5-ht 3) Interacting with opioids receptors 4) Regulating the release of NO
Light et al. (2009); Tchivileva et al. (2010) NCT02437383	Propranolol	Oral administration	Suppress alveolar bone loss and osteoclast hyperactivities	Blockage of β-ARs signal pathway
Ivković et al. (2018)	Estrogen	Oral administration	Reduce hormonal fluctuation related TMD pain	Inhibiting effects caused by abrupt hormonal changes as following: 1) Fibrocartilage degenerative changes 2) Psychophysical symptoms 3) Abnormal Telper 1 and Telper 2 -mediated response
Kızılcık et al. (2017); Zeng et al. (2016) NCT03675659	Magnesium sulfate	Intra-articular injection	Pain relieving	1) Blocking NMDA-depended nerve system activities 2) Suppressing calcium entrance 3) Regulating SOX9 expression 4) Immune modulatory effects
Zotti et al. (2019) NCT03655275	Platelet-rich plasma	Intra-articular injection; Combined with arthrocentesis	Better performance than arthrocentesis alone or combined with HA	1) Promotes cell proliferation and inhibits nuclear factor-κB ligand (RANKL)–induced osteoclast differentiation 2) Promote cartilage matrix production 3) Increasing HA concentration 4) Stabilizing angiogenesis 5) Anti-inflammatory and regenerative effects
Rahimi-Movaghar and Eslami (2012); Daif (2012); Celakil et al. (2019) NCT02997410	Ozone	Ozonized oil/gas injection Noninvasive form	Pain relieving	1) Boosting joint-repairing abilities of fibroblasts 2) Anti-inflammation 3) Chondrogenesis
Christidis et al. (2014); Christidis et al. (2015) NCT02230371	Granisetron	Intramuscular tender-point injections	Pain relieving	5HT-3 selective serotonin receptor antagonist
Thambar et al. (2020)	Botulinum toxin	Intramuscular injections	Controversial	1) Reducing parafunctional motions 2) Analgesic effect for antagonizing the release of substance P, glutamate, and calcitonin gene regulated peptide
Toth et al. (2010); Giacoppo et al. (2015) NCT03994640	Cannabidiol	Transdermal delivery	Pain relieving	1) Acting on the CB2 receptors 2) Binding TRPV1, GPR55 and 5-HT-1A 3) Lowering oxidative and nitrosative stress

### Agents in Clinical trials for TMD Therapy

Clinical trials are essential to verify the toxicity and efficacy of pharmacological agents of TMD. Thus far, nearly a dozen TMD-related clinical trials have been updated in clinicaltrials.gov(https://clinicaltrials.gov/). Most studies were in phase Ⅱ, and the number of participants ranged from dozens to hundreds. In general, several symptoms, including the range of mandibular motion, tenderness of muscle of mastication, and the severity of pain, were tested to evaluate the efficacy of relief, along with the analysis of synovial fluid. Desirable outcomes of clinical trials will provide potent evidence for the extensive use, whereas the detailed mechanism of these drugs should be explored to achieve medication in a precise and accurate manner.

#### Orally Administrated Agents

##### Vitamin B Complex

An intramuscular injection of diclofenac + B vitamin mixture showed a superior analgesic effect compared with the injection of diclofenac alone (Magaña-Villa et al., 2013). A human experiment of patients with knee OA indicated stronger analgesic property of vitamin B than vitamin E and diclofenac; the study highlighted the potential effects of vitamin B on TMD (Dehghan, 2015). Mechanically, the anti-inflammatory and analgesic effect may relate to the cyclooxygenase pathway and opioid receptors (Tamaddonfard et al., 2018). Noticeably, the analgesic effects of B vitamins are not equivalent, with vitamin B12 being more therapeutically beneficial to pain management (Buesing et al., 2019).

##### Propranolol

Propranolol is a racemic mixture in which the S(-)-enantiomer dominates the binding affinity for β-adrenergic receptors (β-ARs). This compound is officially approved for the treatment of conditions, such as cardiac conditions, tremor, migraine, and pheochromocytoma. Emerging clinical evidence indicated that propranolol provides efficacious treatment for the pain management of TMD via the blockage of β-AR signal (Light et al., 2009; Tchivileva et al., 2010). In osteoarthritic joints, evidence of hyperactivities of subchondral bone remodeling and sympathetic nerve fiber sprouting in the osteochondral junction has been displayed (Pongratz and Straub, 2013; Suri and Walsh, 2012). The TMD patients also showed an impaired activation of the sympathetic–adrenergic component of the autonomic nervous system under stress. The elimination of abnormal sympathetic signals by blocking β-ARs will suppress alveolar bone loss and osteoclast hyperactivities (Jiao et al., 2015).

##### Estrogen

TMD prevalence is unequal between genders, with the estimated male to female ratio of patients suffering from TMD at around 1: 3 (Manfredini et al., 2011). Meanwhile, for several female patients suffering from TMD, their menstrual cycles influence the severity of pain systematically as the highest pain occurs during menses and late luteal phase (LeResche et al., 2003). The predisposition and fluctuation of pain can be related to female reproductive hormones, especially estrogen. The effect of estrogen on TMD pain processing is multifaceted. First, estrogen retards fibrocartilage degenerative changes (Robinson et al., 2018). Meanwhile, abrupt hormonal changes can simultaneously cause psychophysical symptoms, possibly exacerbating the pain caused by TMD (Smith et al., 2006). Moreover, the perplexed role of estrogen may relate to their immune and nervous system effects. Reduced and elevated estrogens separately promote T helper 1 (Th-1) and Th-2-mediated response to realized their pro- and anti-inflammation effects (Ivković et al., 2018). The appropriate estrogen regulation across the hormonal cycle works for TMD pain. However, a systematic review evaluating the estrogen effect on TMD in humans revealed that the relationship can be divergent and occasionally contradictory (Berger et al., 2015). A recent review suggested that sex and age-specific estrogen signaling matters when evaluating the effect of estrogen in TMD, and more specific drugs should be developed (Robinson et al., 2020). Thus, studies with larger enrollment of participants can verify the potential of estrogen for general prescription.

#### Agents for IA Injection or Intramuscular Injection

##### Magnesium Sulfate

Magnesium sulfate is a small colorless crystal usually used as an anticonvulsant. Additionally, as an antagonist of N-methyl-D-aspartate (NMDA) receptor, magnesium can modulate pain perception by blocking the nerve system activities that depend on NMDA and achieve the chondrocyte protective effects via decreasing the superfluous entry of extracellular calcium into cells (Srebro et al., 2017). Magnesium sulfate exerts systemic and local immune modulatory effects, potentially relieving the inflammatory pain. For clinical use, IA magnesium sulfate in the knee is suggested as a potential analgesic agent with a low chondrotoxicity and limited side effects. Furthermore, the analgesia effect in patients undergoing surgery with spinal anesthesia has been confirmed (Shah and Dhengle, 2016). Given these information, further exploration of magnesium sulfate in TMD will be worthwhile.

##### Platelet-rich Plasma (PRP)

Consequent pain and motor disturbance in TMD relates to the increased pressure in the joint and to the high amount of cytokines in the synovial liquid (Fernández-Ferro et al., 2017). Sole arthrocentesis of TMJ has achieved satisfying results. Still, researchers continually exploit optimal materials to inject or perform arthrocentesis with PRP, produced by centrifuging heparinized whole autologous blood and separating the platelets from the other blood components, gained extensive attention given that concentrated platelets contain multiple growth factors (GFs). Impressively, PRP not only promotes cell proliferation and cartilage matrix production while inhibiting nuclear factor-κB ligand–induced osteoclast differentiation but also increases the HA and regulate angiogenesis (Hegab et al., 2015; Wang et al., 2018). A latest review based on 10 qualified studies that assessed the effectiveness of PRP use in TMD confirmed that the outcomes of PRP, either by injection or combined with arthrocentesis, exceed that of arthrocentesis alone or combined with HA. Alternatively, the latest generation of PRP, that is, liquid-phase concentrated GF (LPCGF), requires no anticoagulants nor the addition of other agents and possesses a stronger effect than PRP. Researchers indicated that 2 ml LPCGF is highly stable and effective when used to assuage TMD (Yang et al., 2017). PRP prolotherapy is tempting for TMD treatment, given its advantage in regeneration.

##### Ozone

For medical purposes, the potent oxidant, ozone gas, is administered in various forms, such as ozonized oil, besides in gaseous state (Suh et al., 2019). When administered in a joint capsule as a highly reactive molecule, it can boost the joint-repairing abilities of fibroblasts and exert effects, including anti-inflammation and chondrogenesis properties. IA ozone gas injection to treat internal derangement of the TMJ has received recognition. Recently, a noninvasive treatment with improved user experience and positive outcomes was put into use in a device that relies on a glass omega probe to conduct the high-frequency bio-oxidative ozone generated by voltage power.

##### Granisetron

In conditions such as tissue damage or ischemia, the neurotransmitter serotonin 5-HT is released from platelets and mast cells and mediates myalgia and hyperalgesia subsequently by activating the 5-HT-3-receptor. The finding showing the elevated expression of 5-HT-3A-receptor in muscle nerve fibers in women with TMD provides a new therapeutical target (Christidis et al., 2014). Granisetron, a 5HT-3 selective serotonin receptor antagonist, is generally used as an antiemetic and anti-nauseant for cancer chemotherapy patients. Notably, its efficacy as an analgesic was confirmed after repeated intramuscular tender-point injections to patients with chronic TMD (Christidis et al., 2015).

##### Botulinum Toxin (BTX)

BTX is a 150 kDa exotoxin produced by *Clostridium botulinum*, with serotype A (BTX-A) being the most readily available. Once taken by the synapse via receptor-mediated endocytosis, it can cleave synaptosomal-associated protein 25 (SNAP-25) and consequently hamper acetylcholine release from pre-junctional nerves. The inhibition effect on exocytotic activity at the neuromuscular junction would ultimately lead to muscle weakness. Meanwhile, BTX could as well modulate sensory processing for it partially antagonizes the release of substance P, glutamate, and calcitonin gene-regulated peptide (Malgorzata et al., 2017). As for TMD treatment, the capability to reduce parafunctional motions involving the masticatory muscles and analgesic effects may account for BTX’s efficacy. Nevertheless, a latest review provided no clear consensus on the clinical outcomes in the last 30 years. Thus, more tests with high reliability on the therapeutic benefit of BTX for myofascial TMD should be conducted in the future (Thambar et al., 2020).

#### Other Noninvasive Delivered Agents

A wide range of indications, of which pain relief is the most attractive, show that *Cannabis sativa* (CB)-related drugs may hold promise. However, a high potential for abuse and controversial psychoactive properties, presumably mediated by cannabinoid receptors (CB1 and CB2) expressed in the CNS, contributes to its limited applications. For clinical use, researchers performed multiple attempts to achieve the desirable hyperalgesia and allodynia effect while minimizing the side effects. On one side, peripherally restricted CB1 receptor agonists are a plausible solution. No penetration through the BBB of the agents is likely to circumvent their psychoactive effects, according to animal experiments thus far (Yu et al., 2010). On the other side, cannabidiol (CBD) is a non-psychoactive component and has anti-inflammatory and antioxidant properties independent of Δ9-tetrahydrocannabinol (Δ9-THC) (Xu et al., 2020). Evidence shows that CBD can act on the CB2 receptors to inhibit the inflammatory state (Toth et al., 2010), whereas a portion of its effects is realized by binding TRPV1, G protein-coupled receptor 55, and 5-HT-1A other than the CB mechanism. A low level of oxidative and nitrosative stress was also observed due to CBD application (Giacoppo et al., 2015). Clinical trials applying CBD compounded by transdermal delivery in the treatment of painful peripheral neuropathy, and TMD provided positive evidence showing CBD as a promising agent against neuroinflammation and neurodegeneration (Giacoppo et al., 2015). Application of CBD formulation over masseter muscle is also reducing myofascial pain in patients (Nitecka-Buchta et al., 2019b). Furthermore, transdermal delivery may be an effective alternative considering the management of this particular drug.

In the exploration of novel molecules for TMD treatment, researchers exerted efforts in identifying and testing ideal candidates, and several desirable results have been obtained. Typically, traditional or developed drugs that possess anti-inflammation and/or antihypernociception properties dominate the lists of promising candidates. Factors, including inflammatory cytokines, opioid receptors, CO/NO transmitters, and ROS production, are the hotspot of mechanism exploration, which echoes the functions of traditionally used TMD drugs but with less side effects. On the other hand, new targets, including hormone fluctuation, abnormal muscle activities, and psychological factors, provide hints for the use of novel drugs, such as estrogen. Thus, with the additional knowledge on TMD, more promising aspects and targets for drugs and the combination of drugs favoring different mechanisms will probably serve as new and economic medication strategies. From another aspect, the degree of cooperation of patients, which occasionally overrides the efficacy of other drugs, should also be considered when developing new drugs. Novel molecules, such as vitamin B, ozone, BTA, and CBD, can provide better medication experience to patients, considering the “nutrient property” of vitamin B, the long interval of BTA injection, and the noninvasive properties of ozone and CBD application. These drugs can be used to convince patients to accept and stick to the therapy, which will greatly improve the efficacy when the drug itself performs similarly to others. In conclusion, novel molecules developed these years for TMD have been illustrated, and they may serve as a hint for researches to explore further future applications.

## Novel Intra-articular Bioactive Agent Delivery Systems for Treating TMD

As demonstrated above, multiple therapeutic molecules are effective for TMD treatment through noninvasive approaches (i.e. oral analgesics, antidepressants, and NDAIDs) or minimally invasive ones (i.e. intra-TMJ injection of NSAIDs, corticosteroid, HA, and arthrocentesis). However, systematic side effects from oral drugs, such as gastrointestinal damage, cardiovascular risk, and anaphylaxis, and those from frequent intra-TMJ injections, including dizziness, dry mouth, and possible drug addiction, are raising concern. Meanwhile, conventional palliative drug treatments show limitation in the regeneration of chronic and severe TMD (Dashnyam et al., 2018; Donahue et al., 2019). Therefore, novel IA systems, several of which show potential in animal TMD model, that contain biomaterials, cells, and/or bioactive molecules for sustained drug release and TMJ tissue regeneration are attracting attention (Table 3).

**TABLE 3:** Novel intra-articular delivery system in animal TMD models.

	Deliverer type	Material type	Material	Cell	Molecule	Test model	Outcome	Reference	Year
**Delivery system for therapeutic agents**	Hydrogel	Natural	Chitosan+β-GP	-	HA	Rabbit TMJ	Thermosensitive hydrogel releasing HA for over 7 days	Talaat et al. (2016)	2016
	Microparticle	Natural	Gelatin	-	Ibuprofen	Rat TMJ	Microcapsules biocompatible and reduce TMJ OA pain	Kramer et al. (2012)	2012
		Synthetic	PLGA	-	siRNA-PEI	Rat TMJ	Reduced inlammatory expression and nociceptive reaction for 7 days	Mountziaris et al. (2012)	2012
	Nanoparticle	Synthetic	PLGA	-	15 days-PGJ2	Rat TMJ	Decreased IL-1β release and nociceptive behavioral response	Clemente-Napimoga et al. (2012)	2012
		Synthetic	CP + CCT + Pluronic F68	-	naproxen	Rat TMJ	Lipid carriers show reduced IL-1β and TNF-α for over 7 days	Guilherme et al. (2019)	2019
		Synthetic	MSN-CC-PEI	-	HAS2	Rat TMJ	Promoted endogenous HA production and reduced synovial inflammation for 3 weeks	Li et al. (2019)	2019
**Delivery system for regenerative agents**	Hydrogel	Synthetic	Pluronic F-127	Chondrocyte	-	Goat condyle defect	Neocondylar surface covered with fibrocartilaginous tissue	Yu et al. (2011)	2011
		Synthetic	Pluronic F-127	Chondrocyte + BMSC	-	Goat condyle defect	The co-culture system provides chondrogenic environment for BMSC chondrogenic differentiation	Sun et al. (2018)	2018
	Scaffold	Natural	Collagen	Whole BM	-	Rabbit disc defect	Defect healed with new connective tissue in 2 weeks	Kobayashi et al. (2015)	2015
		Natural	Collagen	-	-	Rabbit disc defect	Disc regenerated with normal collagen expression in 3 months	Wang et al. (2017)	2017
		Synthetic	PLGA	BMSC	-	Goat condyle defect	Osteochondral recover within 24 weeks	Zhu et al. (2011)	2011
		Synthetic	PLGA	-	TGF-β1+BMP-2	Rabbit condyle defect	Osteochondral repair after 6 weeks	Dormer et al. (2011)	2011
		Synthetic	PLA	ASC	TGF-β1	Rabbit disc discectomy	Potential system for disc implantation	Ahtiainen et al. (2013)	2013
		Synthetic	PCL + PLGA	-	CTGF + TGF-β3	Rabbit disc defect	Multiphase fibrocartilaginous recovery after 4 weeks	Tarafder et al. (2016)	2016
	Assembled cells	Natural	-	Chondrocyte	-	Minipig disc thinning	Integration with host tissue within 8 weeks	Vapniarsky et al. (2018)	2018

### Novel Delivery Systems for Therapeutic Agents

**FIGURE 3:**
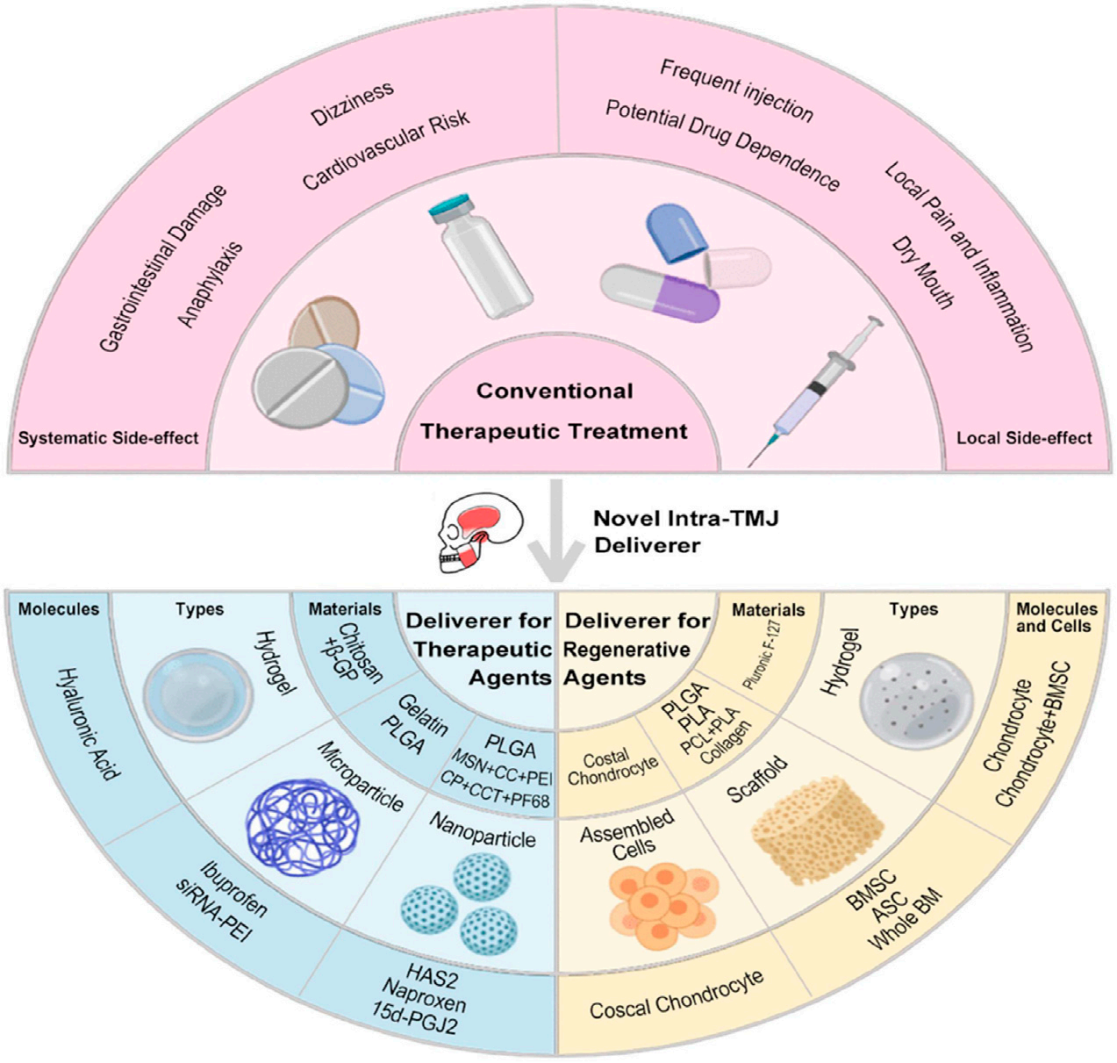
Delivery systems for the therapy of TMD.

Rapid clearance of intra-TMJ injected drugs, such as HA and corticosteroids, requires frequent injections, thus resulting in complications, including infection, fibrous repair, and further joint damage (Dashnyam et al., 2018). Hydrogels and micro- and nanoparticles can assist in sustained therapeutic agent release to address these disadvantages (Figure 3.).

Thermosensitive hydrogels exhibit liquid-to-solid transition capability that is tunable to acquire IA gelation. Chitosan is a biocompatible and biodegradable natural material, whereas β-glycerophosphate (β-GP) is an FDA-approved organic compound. Talaat et al. developed chitosan hydrogels with various β-GP concentrations with *in vivo* gelation time of several minutes. With the incorporation of HA, the hydrogel constantly released the drug in rabbit TMJ for 7 days (Talaat et al., 2016).

Microparticles, with their spherical shapes sized tens to hundreds of micrometers, are suitable to carry agents that interact extracellularly with the host tissue and avoid clearance by phagocytosis (Dashnyam et al., 2018). By contrast, nanoparticles, which are of tens to hundreds of nanometers, possess large surface-to-mass ratio and enter target cells to regulate their fate (Seo et al., 2017).

The natural material gelatin was adopted as a microcapsule to deliver ibuprofen, which showed biocompatibility in rat TMJ and relieved pain from OA (Kramer et al., 2012). In addition, biodegradable synthetic poly (lactic-co-glycolic acid) (PLGA) can be processed into micro- or nanoparticles. For example, PLGA microcapsules loaded with 15 days-PGJ2 decreased IL-1β release and nociceptive behavioral response in rat TMJ (Clemente-Napimoga et al., 2012). Intriguingly, from PLGA microparticles built by Mountziaris et al., the anti-FcγRIII siRNA and the transfecting agent poly (ethylenimine) (PEI) was delivered distinctly, and they were then immediately assembled into nanoscale polyplexes. The system displayed 28 days of controlled release *ex vivo* and alleviation of inflammation and nociceptive reactions in rat for one week (Mountziaris et al., 2012; Mountziaris et al., 2010). In addition, Guilherme and colleagues established a nanostructured lipid carrier with the mixture of cetyl palmitate and capric/caprylic triglycerides and the cooperation of Pluronic F68. The deliverer contributed to the administration of naproxen, reducing the IL-1β and TNF-α levels in rat TMJ for over 7 days (Guilherme et al., 2019). Furthermore, mesoporous silica nanoparticles with a core–cone structure were engineered by Li et al., (2019). With proper pore size and PEI functionalization, the particles were utilized to deliver hyaluronan synthase type 2 directly into rat TMJ synoviocytes. Consequently, the endogenous HA production was boosted, with synovial inflammation being inhibited for 3 weeks.

### Novel Delivery Systems for Regenerative Agents

For the severe types of TMD, such as internal derangement, disc thinning, disc perforation, and osteochondral damage of condyle, regenerative tissue engineering approaches are in demand. Such systems typically consist of bioactive agents, including scaffold, cells, and/or GFs (Donahue et al., 2019; Aryaei et al., 2016; Acri et al., 2019).

In animal models of TMJ damage, natural collagen (Kobayashi et al., 2015; Wang et al., 2017), synthesized PLGA (Zhu et al., 2011; Dormer et al., 2011), polylactic acid (Ahtiainen et al., 2013), and polycaprolactone (PCL) (Tarafder et al., 2016) were fabricated into scaffolds, whereas Pluronic F-127-based hydrogel supported regenerative cell implantation (Yu et al., 2011; Sun et al., 2018). For cells in the systems, articular chondrocytes, costal chondrocytes, whole bone marrow, bone marrow stem cells (BMSCs), and adipose stem cells (Ahtiainen et al., 2013) were adopted. Soluble agents were added to the cell culture to modify the biological and physical properties of cells. For instance, NELL-1 was used in the pre-treatment of BMSCs to enhance their chondrogenic differentiation (Zhu et al., 2011). Meanwhile, costal chondrocytes cultured with chondroitinase ABC showed increased tensile ability (Vapniarsky et al., 2018). In regard to the bioactive molecules delivered, the transforming GF (TGF)-β family (Dormer et al., 2011; Ahtiainen et al., 2013; Tarafder et al., 2016), connective tissue growth factor (CTGF), and bone morphogenetic protein-2 were harnessed to regulate the growth and function of the introduced cells and host tissue.

On the other hand, the scaffold-free approaches for regenerative systems are also attractive because they present no problems compared with their scaffold-based counterparts, whose scaffolds may degrade faster than the formation of new tissue or wreaked by toxic degraded polymers (Donahue et al., 2019; Acri et al., 2019). With the passaged, redifferentiated, and self-assembled costal chondrocytes delivered orthotopically as scaffold-free implant, Vapniarsky et al. successfully regenerated the thinned minipig TMJ disc. The material showed gradual amalgamation with the host tissue within 8 weeks (Vapniarsky et al., 2018).

Furthermore, the advancement of fabrication technology strengthened the development of delivery systems. As an example, three-dimensional printing features custom-designed structural and physical properties. Utilizing the different melting temperatures of PLGA and PCL, Tarafder and colleagues encapsulated CTGF and TGF-β3 in PLGA microspheres, which were then embedded in PCL microfibers. Finally, they established a micro-precise anisotropic scaffold and witnessed the multiphased fibrocartilage recovery of rabbit TMJ disc after 4 weeks (Tarafder et al., 2016).

### Hurdles and Prospects of Intra-TMJ Delivery Systems to Treat TMD

Attempts to develop intra-TMJ delivery systems in animal models are emerging but still limited to several studies thus far. Nevertheless, various novel designs, which have the potential to be applied to IA TMD treatment, are reported in *in vitro* studies for TMJ and in research on other joint disorders (Dashnyam et al., 2018; Donahue et al., 2019). Importantly, recent studies raised quotable ideas to facilitate the improvement of current intra-TMJ delivery systems. First, the advanced agents for administration, such as ion/drug co-delivery (Seo et al., 2017) and antioxidants, can be incorporated (Dashnyam et al., 2018). Second, novel materials can also be employed. For instance, TMJ disc cells are viable and expressed in the matrix of titanium dioxide (TiO_2_) nano thin films (Ronald and Mills, 2016). Given the potential of TiO_2_ for layer-by-layer nanoassembly (Kommireddy et al., 2006), TiO_2_ materials can be developed into deliverer scaffolds. Third, new sources of cells can be adopted, such as Wharton’s jelly-derived mesenchymal stem cells, dental pulp stem cells, nucleus pulposus cells, and induced pluripotent stem cells. Fourth, the optimization of physical properties and incorporation of biophysical stimuli can improve the delivery efficacy (Dashnyam et al., 2018; Donahue et al., 2019; Acri et al., 2019). Finally, preclinical models of large mammals may well mimic TMD pathogenesis in humans and thus promote the bench-to-bedside transition of novel intra-TMJ therapies (Almarza et al., 2018).

## Future Perspectives

Over the past 25 years, agents for treating TMD and relative syndromes have evolved from commonly used small molecules to a dynamic field of macromolecules, natural products, and novel functional materials. The translation of novel therapeutic strategies has been greatly inspired by tissue engineering based on the understanding of pathological mechanism of TMD. This field has achieved considerable progress in the satisfied management of pain and inflammatory syndromes and new attempts in the prevention of TMD development by regenerative medicine. As a result of these efforts, growing evidence indicates that adaptive reconstruction of TMJ can be effectively applied in preclinical and clinical studies, which may finally induce satisfied clinical outcomes. Eventually, therapeutic approaches may enable a more effective and personalized therapy for TMD patients.

## Author Contributions

Conceptualization, MJW and MRW; writing and original draft preparation, MJW, JC, YY, SH, and YW; writing and review and editing, MRW; visualization, JC, YY, and MRW; project administration and funding acquisition, MJW. All authors have read and agreed to the published version of the manuscript.

## Funding

This research was supported by Natural Science Foundation of China grant numbers: 81970956, 81900806; Zhejiang Qianjiang Talent Project grant number: QJD1902024.

## Conflict of Interest

The authors declare that the research was conducted in the absence of any commercial or financial relationships that could be construed as a potential conflict of interest.
